# Antimicrobial resistance and molecular characteristics of bovine mastitis-associated methicillin-resistant *Staphylococcus aureus*: potential for cross-species transmission of ST59-MRSA

**DOI:** 10.1128/spectrum.02800-24

**Published:** 2025-05-21

**Authors:** Wen Sun, Jiayi Liu, Shuangshuang Li, Xiaoman Zhu, Xiangyun Wu, Baojing Dou, Xiaomin Pang, Keke Tian, Peipei Wang, Haihong Hao, Yulian Wang

**Affiliations:** 1 College of Animal Science and Veterinary Medicine, Huazhong Agricultural University 47895, Wuhan, Hubei, China; 2 National Reference Laboratory of Veterinary Drug Residues, Huazhong Agricultural University 47895, Wuhan, Hubei, China; 3 Shenzhen Kingsino Technology Co., LTD, Shenzhen, China; DePauw University, Greencastle, Indiana, USA

**Keywords:** livestock-associated *Staphylococcus aureus*, bovine mastitis, multidrug-resistant, molecular characteristics, ST59

## Abstract

Mastitis caused by methicillin-resistant *Staphylococcus aureus* (MRSA) is a common issue in dairy farming, with sequence types (STs) related to cows mainly including ST9 and ST97. ST59, the predominant community-acquired clone, is still less reported in dairy cows. This study investigated the antimicrobial resistance patterns and molecular characteristics of 77 *Staphylococcus aureus* isolates obtained from eight dairy farms in the mid-east of China during 2019–2020, focusing particularly on the bovine mastitis-related livestock-associated MRSA (LA-MRSA) clone ST59. Among the 77 isolates, 14 isolates were identified as MRSA. A total of 20 STs were identified, with ST59 being the most prevalent among MRSA isolates (35.7%). All MRSA isolates possessed various Staphylococcal cassette chromosome *mec* (SCC*mec*) types, including XII (*n* = 5), IV.a (*n* = 4), IV.c (*n* = 2), IV.g (*n* = 2), and V (*n* = 1). Three MRSA lineages were identified: MRSA-ST59-t437-SCC*mec* IV.a/IV.g (*n* = 5), MRSA-ST9-t899-SCC*mec* XII (*n* = 4), and MRSA-ST88-t3622-SCC*mec* IV.c (*n* = 2). Approximately 44.2% of isolates demonstrated multidrug resistance. MRSA isolates showed a higher prevalence of antimicrobial resistance compared to methicillin-sensitive *Staphylococcus aureus* (MSSA) isolates. Virulence factor assays revealed that all MRSA isolates carried at least hemolysin genes and enterotoxin genes. ST59-MRSA strains showed the closest genetic relationship with human-derived strains, indicating a potential public health risk due to transmission of *Staphylococcus aureus* between livestock and humans. This study highlights the significant prevalence of the bovine mastitis-related LA-MRSA clone ST59 in the mid-east of China. Therefore, reinforcing monitoring and implementing preventive measures are essential to combat LA-MRSA.

**IMPORTANCE:**

Obtained the prevalence and molecular characteristics of *Staphylococcus aureus* in dairy farms in the mid-east of China from 2019 to 2020. Recently identified livestock-associated methicillin-resistant *S. aureus* (LA-MRSA) clones in cattle, including ST59-MRSA, may have originated from human sources, suggesting a potential risk for interspecies transmission.

## INTRODUCTION

*Staphylococcus aureus* (*S. aureus*) is a major contributor to bovine mastitis, with methicillin-resistant *S. aureus* (MRSA) posing significant risks to both human and animal health (1). MRSA infections are often linked to specific clones prevalent in certain regions (2). In China, ST59 is a predominant community-acquired clone and has been occasionally reported in dairy farms (3, 4). Of particular concern is the potential transmission of MRSA between humans and animals. Livestock-associated MRSA (LA-MRSA) is believed to circulate between humans and animals through the food chain or farm environment.

Despite the widespread use of antibiotics to treat bovine mastitis, numerous studies have demonstrated that *S. aureus* tends to develop resistance to a broad spectrum of antibiotics, particularly β-lactams (5–8). MRSA is of particular concern due to its greater antimicrobial resistance compared to methicillin-sensitive *S. aureus* (MSSA). Recent studies indicate a global MRSA prevalence of 4.30%, with the prevalence in Asia reaching 6.47%, exceeding the global average (9). This higher prevalence may be attributed to the overuse of antibiotics and the lack of efficient regulatory frameworks in low-income countries within Asia.

The development of antimicrobial resistance and the presence of virulence factors are critical factors in the transmission and pathogenesis of *S. aureus* (10). Resistance in *S. aureus* arises from mutations or the acquisition of specific antibiotic-resistant genes, which can be easily transferred between hosts and exist in various combinations within different strains. Some strains may harbor two or more resistance genes for the same class of antibiotics (6). *S. aureus* harbors multiple virulence factors, including hemolysins, leukocidins, exfoliative toxins, enterotoxins (SEs), toxic shock syndrome toxin 1, and the ability to form biofilms. SEs, particularly classical SEs (SEA to SEE) and newer SEs (SEG-SElY), play a significant role in food contamination, with SEA being the most common (11, 12). Antibiotic treatment can also affect the expression of certain virulence factors. For example, β-lactam therapy can induce the production of α-toxin, with MRSA producing more α-toxin than MSSA post-treatment (13). However, clindamycin and erythromycin have been shown to inhibit α-toxin production (5, 14).

The aim of this study was to systematically investigate the prevalence, antimicrobial resistance, and virulence characteristics of *S. aureus* strains in the mid-east of China and to conduct a phylogenetic analysis of ST59-MRSA isolates.

## RESULTS

### Molecular types of *Staphylococcus aureus* isolates

**Fig 1:**
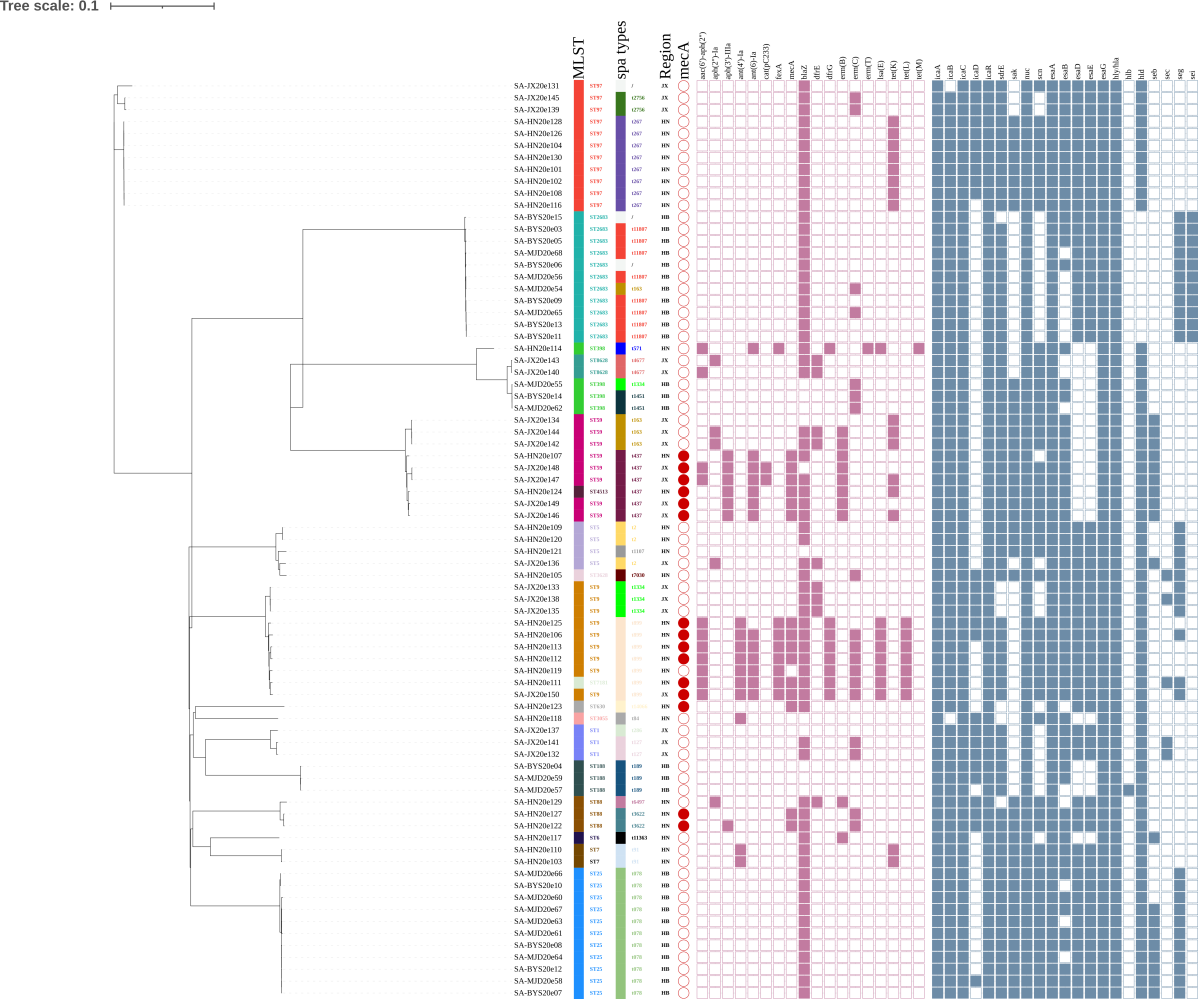
Phylogenetic analysis of 77 *S*. *aureus* strains. Different colored bar patterns represent different sequence types and *spa* types. HB, Hubei Province; HN, Hunan Province; and JX, Jiangxi Province. The red circular pattern indicates strains that harbored the *mecA* gene. ARGs and VFs: the filled squares (purple or blue) indicate the presence and empty squares the absence of individual genes indicated at the head of each column.

**Fig 2:**
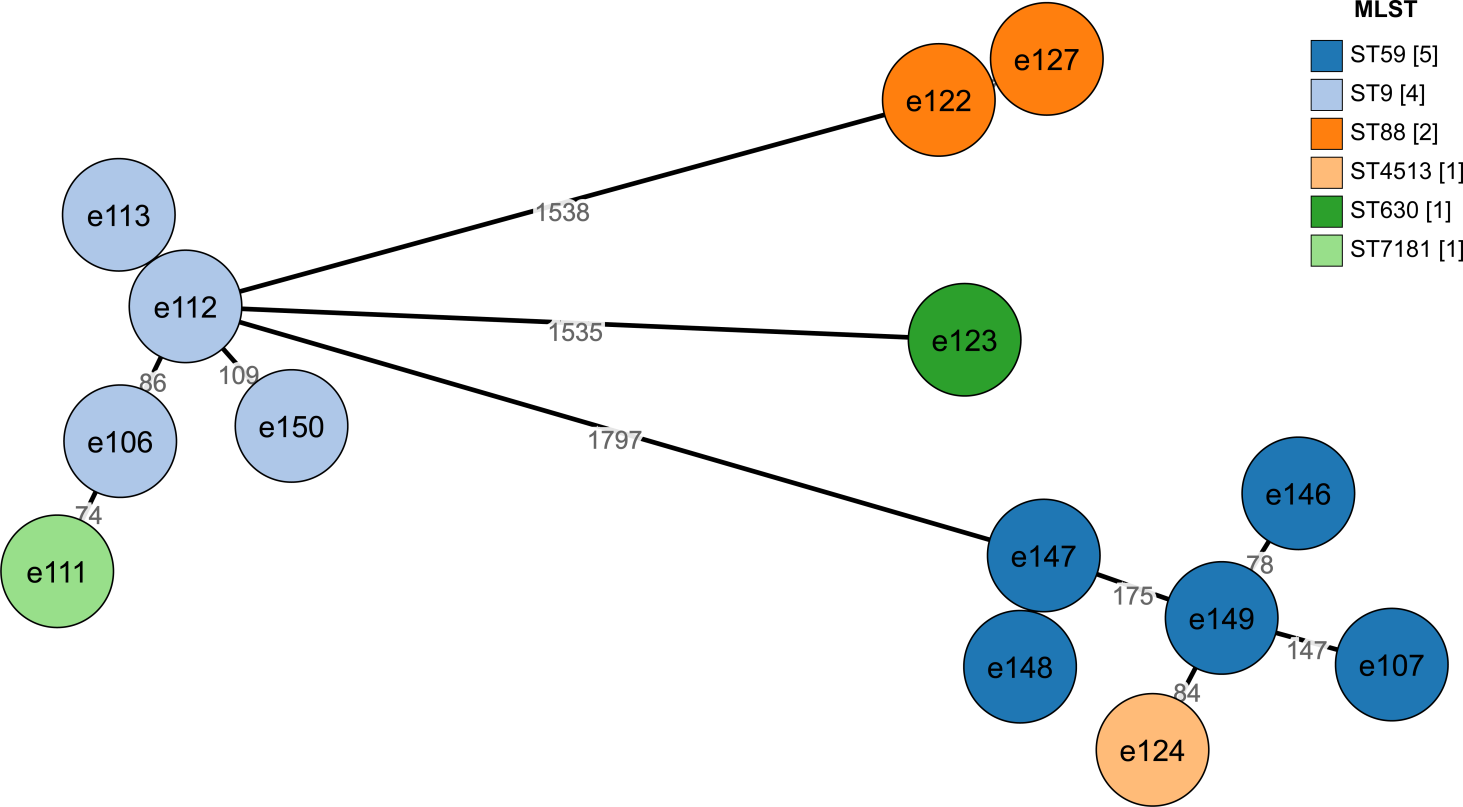
MST of 14 MRSA isolates. Each circle represents a strain, with colors corresponding to the legend STs, and distances labeled on each branch.

A total of 77 *S*. *aureus* strains (21.2%, 77/364) were isolated from dairy farms across three provinces in China (Hubei, Hunan, and Jiangxi), including 14 MRSA strains (18.2%, 14/77). Additionally, one isolate (SA-HN20e125) was found to harbor the *mecA* gene but was sensitive to oxacillin and was therefore classified as an MSSA strain. The isolates comprised 18 distinct sequence types (STs), with 15 STs belonging to 10 clonal complexes (CCs). Analysis of multilocus sequence typing (MLST) data indicated that CC97-ST973 and CC2683-ST2683 were the most frequent (14.3%, *n* = 11), followed by CC1-ST9 (11.7%, *n* = 9), CC59-ST59 (10.4%, *n* = 8), among other lineages. Furthermore, 23 distinct *spa* types were identified, with t078 (14.3%, *n* = 11) being the most common (Fig. 1). Among the MRSA isolates, six STs were identified: ST59 (35.7%, *n* = 5), ST9 (28.6%, *n* = 4), ST88 (14.3%, *n* = 2), ST630 (7.1%, *n* = 1), ST4513 (7.1%, *n* = 1), and ST7181 (7.1%, *n* = 1). Staphylococcal cassette chromosome *mec* (SCC*mec*) typing revealed three types (IV, V, and XII) among 14 MRSA isolates. Type IV was the most predominant type (57.1%, *n* = 8), found in ST59, ST88, and ST4513. By combining ST, *spa* type, and SCC*mec*, three main MRSA lineages were identified: MRSA-ST59-t437-SCC*mec* IV.a/IV.g (*n* = 5), MRSA-ST9-t899-SCC*mec* XII (*n* = 4), and MRSA-ST88-t3622-SCC*mec* IV.c (*n* = 2). A minimum spanning tree constructed for the MRSA isolates delineated their evolutionary relationships, revealing that ST7181 is closely related to ST9, and ST4513 is closely related to ST59 (Fig. 2).

### Antimicrobial resistance phenotypes and antimicrobial resistance genes

**Fig 3:**
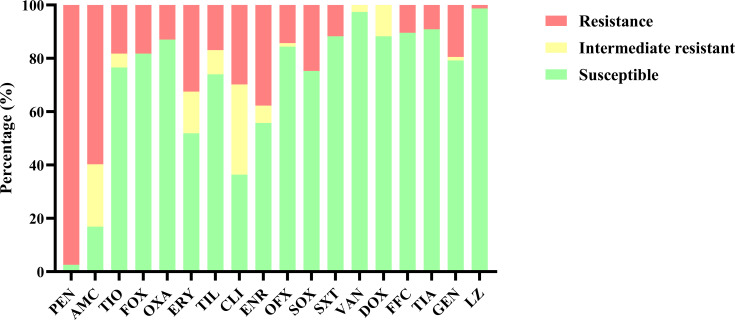
Antimicrobial susceptibility tests of 77 *S*. *aureus* strains. PEN, penicillin; AMC, amoxicillin/clavulanic acid; TIO, cftiofur; FOX, cefoxitin; OXA, oxacillin; ERY, erythromycin; TIL, timicosin; CLI, clindamycin; ENR, enrofloxacin; OFX, ofloxacin; SOX, sulfisoxazole; SXT, sulfamethoxazole; VAN, vancomycin; DOX, doxycycline; FFC, florfenicol; TIA, tiamulin; GEN, gentamicin; and LZ, linezolid.

The *S. aureus* isolates demonstrated resistance to most antibiotics; however, resistance to doxycycline was not observed in any isolates. Ninety-nine percent of the isolates were resistant to multiple antibiotics, with 44.2% classified as multidrug-resistant (MDR) strains (Fig. 3). Compared to MSSA strains, MRSA strains exhibited higher resistance rates to most antibiotics (88.9%). MRSA isolates were entirely resistant (100%) to penicillin, amoxicillin-clavulanic acid, and cefoxitin, and the resistance rates of MRSA to other antimicrobials ranged from 7.1% to 92.9%. However, it remains sensitive to vancomycin and doxycycline, indicating that these two antibiotics can still be used as alternative treatments (Table S2). A total of 18 antimicrobial resistance genes were identified (Table S3). The profiles of resistance genes in the 14 MRSA isolates are presented in Table S5. All MRSA isolates tested positive for the *mecA* gene. Additionally, 85.7% of MRSA isolates harbored one of the *erm* genes, predominantly *erm*(C) (*n* = 7) and *erm*(B) (*n* = 6). Eight MRSA isolates harbored tetracycline resistance genes, including *tet*(L) (*n* = 5) and *tet*(K) (*n* = 3). The *erm*(B) gene, linked to macrolide resistance, was primarily observed in ST59-MRSA isolates (*n* = 5). ST9 and ST7181 typically harbored more resistance genes than ST59, ST88, ST630, and ST4513, likely due to differences in their SCC*mec* elements.

Looking at the correlation between phenotypic and genotypic resistance profiles, specifically, 100.0% (8/8) of the isolates resistant to florfenicol carried the *fexA* gene, and the presence of this gene was significantly associated with florfenicol resistance (*P* < 0.001, χ² = 77.00). Similarly, significant associations were found between the carriage of the *aac(6′)-aph(2′′*), *ant(4′)-Ia*, *ant (6)-Ia*, and *aph(2′′)-Ia* genes and resistance to aminoglycoside antibiotics, with these genes being present in 33.3% to 66.7% of aminoglycoside-resistant isolates (*P* < 0.001) (Table 1). Notably, certain strains possess tetracycline resistance genes, specifically *tet*(K) and *tet*(L), yet they do not exhibit resistance to tetracycline antibiotics. On the other hand, we have identified some isolates as harboring phenotypically susceptible resistance genes.

**TABLE 1:** The association between phenotypic and genotypic antibiotic resistance profiles of *S. aureus* isolates

Drug group and resistance genes		Number of isolates carrying the resistance gene		*χ* ^2^	*P*-value
		Resistant^*a*^	Non-resistant^*b*^		
Aminoglycosides	*aac(6')-aph(2''*)	10 (66.7%)	1 (1.6%)	41.74	<0.001
	*ant(4')-Ia*	7 (46.7%)	3 (4.8%)	18.70	<0.001
	*ant(6)-Ia*	9 (60.0%)	4 (6.5%)	24.68	<0.001
	*aph(2'')-Ia*	5 (33.3%)	0 (0.0%)	22.10	<0.001
	*aph(3')-IIIa*	2 (13.3%)	5 (8.1%)	0.40	0.524
Amphenicols	*cat(pC233*)	0 (0.0%)	2 (2.9%)	0.38	0.626
	*fexA*	8 (100.0%)	0 (0.0%)	77.00	<0.001
β-lactams	*blaZ*	66 (88.0%)	0 (0.0%)	12.32	<0.001
	*mecA*	15 (20.0%)	0 (0.0%)	0.49	0.481
Folate pathway antagonist	*dfrE*	2 (22.2%)	7 (10.3%)	1.09	0.295
	*dfrG*	2 (22.2%)	6 (8.8%)	1.53	0.216
Lincosamides	*erm*(B)	9 (39.1%)	1 (1.9%)	19.84	<0.001
	*erm*(C)	8 (34.8%)	10 (18.5%)	2.38	0.557
	*lsa*(E)	8 (34.8%)	0 (0.0%)	20.96	<0.001
Macrolides	*erm*(B)	9 (39.1%)	1 (1.9%)	19.84	<0.001
	*erm*(C)	11 (47.8%)	7 (13.0%)	10.95	0.001
	*erm*(T)	1 (4.3%)	0 (0.0%)	2.38	0.123

^*a*^ Isolates that are phenotypically resistant to the indicated antibiotic and harbor the resistance gene(s).

^*b*^ Isolates phenotypically susceptible or intermediately resistant to the indicated antibiotic but harbor the resistance gene(s).

### Distribution and associations of virulence genes

**Fig 4:**
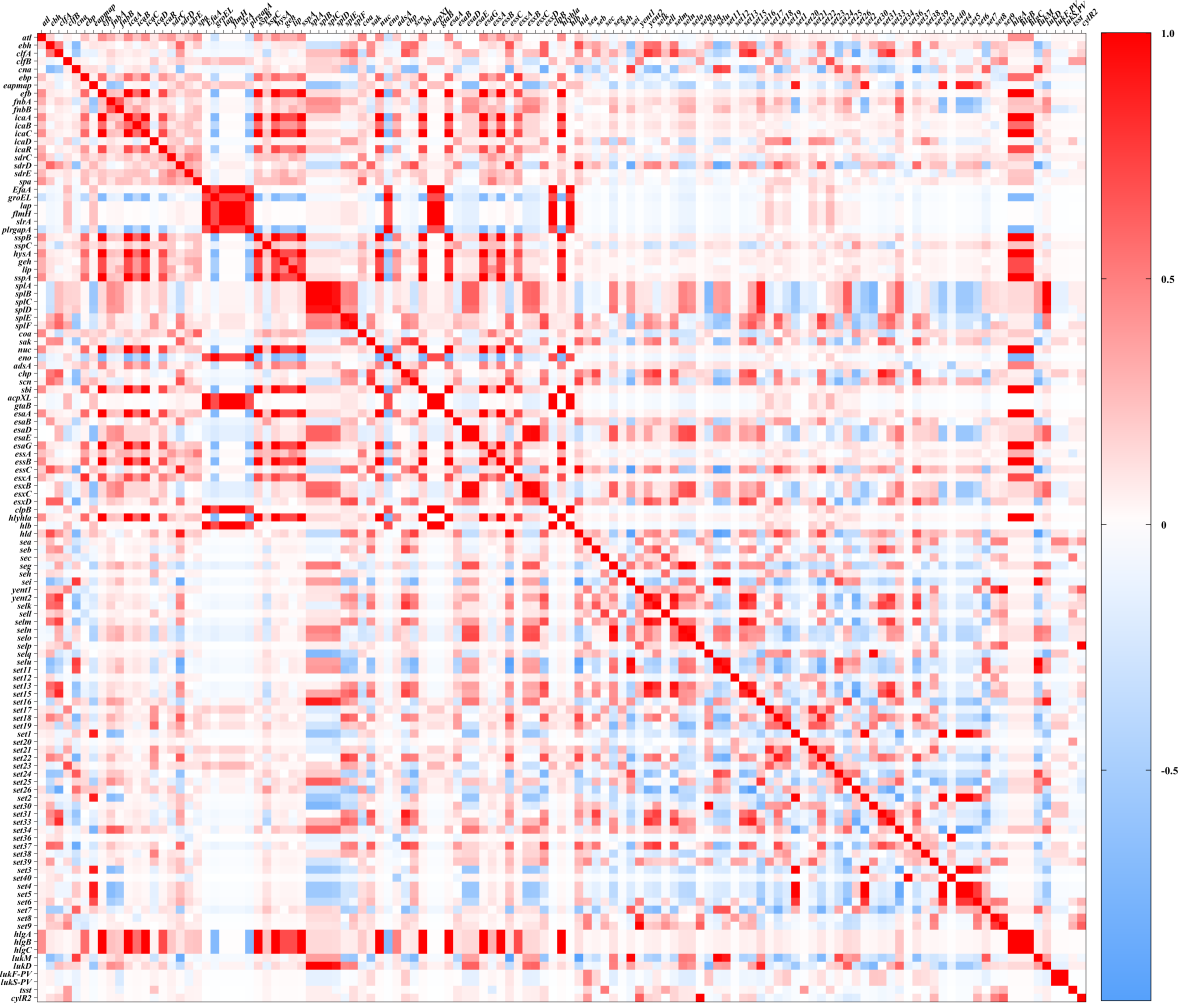
Pairwise associations of the virulence gene in 77 *S*. *aureus* strains. Associations were computed using the Pearson coefficient. Color represents the types of associations (red, positive; blue, negative). The color intensity reflects the significant correlation.

One hundred twenty-one virulence genes were screened from 77 *S. aureus* isolates (Table S4). The associations among these virulence genes were analyzed using heatmaps (Fig. 4). Most associations were neutral or very weak, though significant positive and negative correlations were identified among various gene groups. Specifically, the serine protease genes *splA*, *splB*, *splC*, *splD*, *splE*, and *splF* exhibited positive correlations with one another, as well as with the type VII secretion system genes *esxA*, *esxB, esxC*, and *esxD*. Additionally, distinct patterns of association were noted among toxin genes. Classical enterotoxin genes *sea*, *seb*, and *sec* demonstrated positive correlations with hemolysin genes *hly*/*hla* (alpha-hemolysin gene) and *hld* (beta-hemolysin gene), while exhibiting strong negative correlations with the hemolysin gene *hlb* and leukotoxin genes *lukD* and *lukM*. All MRSA isolates carried hemolysin genes, including *hly*/*hla*, *hld*, *hlgA* (gamma-hemolysin chain II precursor gene), *hlgB* (gamma-hemolysin component B precursor gene), and *hlgC* (gamma-hemolysin component C gene). Two ST88-MRSA isolates harbored leukocidin component genes *lukD*, while one ST59-MRSA isolate carried Panton-Valentine leukocidin genes *lukF-PV* and *lukS-PV*. The majority of MRSA isolates, with the exception of two ST9-MRSA strains, possessed at least one gene associated with staphylococcal enterotoxins (SE). The following SE genes were identified: *sea* (*n* = 5), *seb* (*n* = 6), *sec* (*n* = 1), and *seg* (*n* = 3) (Table S5).

### Phylogenetic construction of *Staphylococcus aureus* ST59

**Fig 5:**
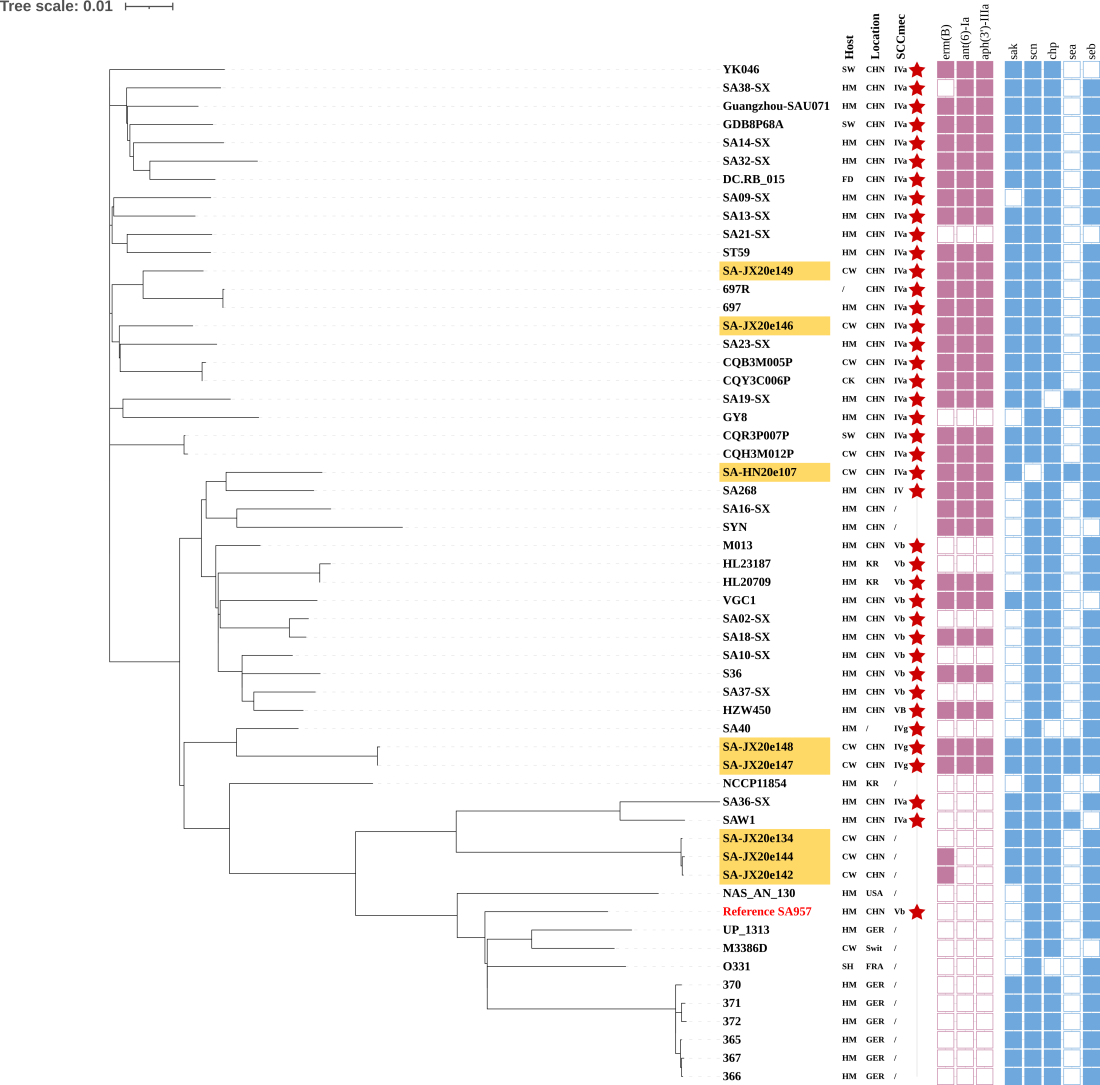
Phylogenetic tree of ST59 *S. aureus*. The ST59 isolates in this study have an orange background color. Hosts: FD, food; HM, human; SW, swine; SH, sheep; CW, cow; CK, chicken; /, others. Locations: CHN, China; FRA, France; USA, United States of America; GER, Germany; Swit, Switzerland; KR, Korea; /, unknown. The red star pattern represents harbored the *mecA* gene. ARGs and VFs: the filled squares (purple or blue) indicate the presence and empty squares the absence of individual genes indicated at the head of each column.

To explore the evolution of ST59 isolates, we constructed a phylogenetic tree with 8 ST59 isolates in this study and 48 ST59 isolates from the NCBI database (Fig. 5). The analysis revealed that the eight ST59 isolates from this study could be categorized into different clades, which may be related to their origins from different farms. The MRSA strain SA-HN20e107 exhibited a close genetic relationship to the human MRSA isolate SA268 from Zhejiang Province, with only 308 single-nucleotide polymorphism (SNP) loci differences. Conversely, strains SA-JX20e146 and SA-JX20e149 were more closely related to human isolates from Shanxi Province (SA21-SX and SA23-SX). The phylogenetic analysis depicted the close genetic relationship between ST59-MRSA isolates and human-derived isolates, suggesting potential transmission dynamics between humans and cows (Fig. 4; Table S6).

## DISCUSSION

*S. aureus* is the primary pathogen responsible for bovine mastitis, a prevalent affliction in dairy herds (15). The overuse of antibiotics has led to the emergence of MDR strains, particularly MRSA, posing significant challenges for effective treatment. Vigilant surveillance of *S. aureus* prevalence, especially MRSA, is crucial for mitigating associated risks (16). The contiguous nature of the three provinces and documented cattle transportation networks likely contribute to the observed regional dissemination of *Staphylococcus aureus*. Our findings of identical STs and congruent antibiotic resistance profiles across different provinces align with previous reports demonstrating MSSA/MRSA transmission via livestock movement in geographically proximate areas (17). β-lactam antibiotics, particularly penicillin, are widely used in dairy cows globally (18, 19). Alarmingly, 98.0% of isolates in this study were resistant to penicillin, a rate similar to that observed in Shanghai (96.7%) (20) and higher than the resistance rates reported for *S. aureus* strains isolated from milk and dairy products worldwide (21). Furthermore, MRSA isolates in this study were resistant to a variety of antibiotics, consistent with previous research findings (22, 23).

The 14 MRSA isolates in this study include five SCC*mec* XII strains (four ST9-t899 and one ST7181-t899), eight SCC*mec* IV strains (five ST59-t437, two ST88-t3622, and one ST4513-t437), and one SCC*mec* V strain (ST630-t14066). These isolates belong to different *spa* and SCC*mec* types, indicating their genetic diversity. ST9 is the predominant LA-MRSA clonal lineage in Asian pigs, exhibiting various SCC*mec* types across different regions of Asia (24). In China, SCC*mec* III is the most prevalent, though SCC*mec* XII, a novel SCC*mec* type, was first identified in two ST9-t899-MRSA isolates in China (25). The presence of ST9-SCC*mec* XII-t899 in both pigs and cows suggests potential for interspecies transmission. LA-MRSA is widely distributed among various animal species, particularly affecting both humans and livestock (26). In this study, ST59-MRSA (35.7%) was the dominant MRSA type, belonging to the epidemic community-associated MRSA clone. This clone is well-documented for its association with humans (27–29). All ST59-MRSA isolates originated from mastitis cases. Recent research shows that the community-associated CC59-ST59-t437-IV lineage is prevalent in China (30). Consequently, it is plausible that ST59-MRSA could be transmitted to dairy farms via environmental factors or human carriers. However, there is currently no strong evidence to prove that ST59-MRSA isolates can spread between animals or from animals to humans, necessitating further validation and research.

Among the 77 *S. aureus* isolates, the prevalence of MSSA was 81.8%. In addition, 15 distinct STs were identified in MSSA, but only six were found in MRSA. The most frequently represented lineages in MSSA were CC97, CC1, and CC2683, accounting for 17.5% (11/77) of the isolates. A recent study on bovine mastitis infection across six provinces in China from March 2010 to August 2013 also identified CC97 as the dominant clonal lineage (6). However, the difference is that all CC97 strains in this study were MSSA.

The emergence and spread of antimicrobial resistance genes are crucial factors in the development of antibiotic resistance. Resistance to florfenicol in Staphylococcus is primarily mediated by the genes *fexA*, *fexB*, *cfr*, and *optrA* (31). Consistent with this, all isolates harboring the *fexA* gene (100%, 8/8) demonstrated resistance to florfenicol, further supporting this observation. Notably, all ST9-MRSA harbored the diaminopyrimidine resistance gene *dfrG*, which is associated with trimethoprim resistance in human infections (32). Although certain *S. aureus* strains harbored tetracycline resistance genes, they did not exhibit resistance. This discrepancy may result from factors such as insufficient gene expression, gene inactivation, lack of synergy with other resistance mechanisms, or other unidentified elements. These findings underscore that the presence of resistance genes does not always correlate with phenotypic resistance, highlighting the complexity of resistance expression in bacterial strains.

The production of hemolysin by *S. aureus* caused infections in cows. All *S. aureus* strains in this study carried at least one hemolysin-coding gene. However, the prevalence of the *hly/hla* gene was significantly higher than that of the *hlb* gene, reflecting trends observed in human clinical isolates (33). Enterotoxin is intricately linked to food safety. Peles et al. found that the enterotoxin genes *seg* and *sei* were exclusively present in strains from mastitic milk, consistent with our findings (34). The classical SE genes *sea* and *seb* were present in isolates of the clonal lineages CC5, CC59, and CC25. The non-classical SE gene *seg* was prevalent in the clonal lineages CC1, CC25, and CC5. The low prevalence of the *sec* gene (4.7%) and the high prevalence of the *seg* gene (61.7%) are believed to be connected to a decreased likelihood of intramammary infections in cows (35–37). Both classical and novel enterotoxins pose health risks; thus, monitoring of *S. aureus* enterotoxins cannot be ignored (38).

### Conclusions

This study highlights the presence of MRSA in dairy cows, which always exhibited multidrug resistance. High-risk LA-MRSA ST59 lineages were detected at dairy farms, indicating a potential public health risk of transmission of *S. aureus* between livestock and humans. Therefore, it is necessary to strengthen the continuous monitoring of animals and breeders on dairy farms.

## MATERIALS AND METHODS

### Isolation and identification of *Staphylococcus aureus* strains

In 2019 and 2020, a total of 364 milk samples were randomly collected from eight commercial dairy farms in Hubei, Hunan, and Jiangxi Provinces. It is essential to note that the three provinces are contiguous, forming a geographically integrated region. This adjacency facilitates frequent cross-provincial movements of cows between dairy farms, which may influence pathogen dissemination dynamics. The number of lactating cows on farms A, B, C, D, E, F, G, and H was 202, 1,200, 400, 272, 287, 250, 2,850, and 1,100, respectively. The milk samples were obtained from cows that exhibited no signs of clinical or subclinical mastitis, as well as from those diagnosed with clinical mastitis and subclinical mastitis. Clinical mastitis is characterized by symptoms such as swollen and painful udders and elevated body temperature, whereas subclinical mastitis is identified by a somatic cell count exceeding 500,000 cells/mL, without any apparent pathological changes, in accordance with the Chinese agricultural industry standard NY/T 2692-2015 (National Standard of the People’s Republic of China, 2015). Veterinary care provided on these farms included daily monitoring and the administration of antibiotics. A variety of antibiotics were utilized, primarily amoxicillin/clavulanic acid, ceftiofur, florfenicol, gentamicin, and tilmicosin, as part of the established prevention and treatment protocols.

Prior to milk collection, udders were thoroughly washed and dried, followed by disinfection using cotton balls saturated with 75% ethanol. All samples were collected aseptically and placed into sterile, non-heat-source sampling bags, which were then promptly transported to the laboratory for further processing. In the laboratory, samples were initially enriched in 7.5% sodium chloride broth. Subsequently, they were cultured on BP agar plates (Haibo Biotech, Qingdao, China) and identified as *S. aureus* based on positive results from the catalase and rabbit plasma coagulase tests. Colonies exhibited a characteristic purple color on the chromogenic medium. To confirm identification, polymerase chain reaction amplification of the *S. aureus nuc* gene (forward primer: GGCAATACGCAAAGAGGTT; reverse primer: CGTTGTCTTCGCTCCAAAT) and sequencing of the 16S rDNA (forward primer: AGAGTTTGATCCTGGCTCAG; reverse primer: GGTTACCTTGTTACGACTT) were performed. *S. aureus* ATCC 29213 was employed as a positive control strain, with experimental procedures adapted from El Zowalaty et al. (39). Positive strains were then mixed with 40% glycerol in a 1:1 ratio and stored at −80°C for further analysis.

### Genomic DNA extraction and whole genome sequencing

DNA extraction was performed using the *SteadyPure* Bacteria Genomic DNA Extraction Kit (Accurate Biotechnology Co. Ltd., Hunan, China). Two-terminal sequencing was conducted on the Illumina Novaseq-PE150 sequencing platform. The genome library was constructed and sequenced following quality control procedures. The sequencing reads were cut and cleared by FastQC and AdapterRemoval. The sequences were then assembled using SPAdes (v3.15.5) (https://github.com/ablab/spades), and genome annotation was performed using Prokka. SCC*mec* types and *spa* types of all isolates were predicted using the SCC*mec*Finder tool and *spa*Typer tool available in MLST 2.0 (https://www.genomicepidemiology.org/services/).

### Multilocus sequence typing

The MLST method was utilized to assess the genetic diversity of MRSA isolates. Whole genome sequencing (WGS) data were submitted to the PubMLST website (https://pubmlst.org/) to determine STs, based on seven housekeeping genes: *aroE*, *arcC*, *glpF*, *yqiL*, *tpi*, *gmk*, and *pta*. To illustrate the genetic relationships among the isolates, a minimum spanning tree was generated using the Grapetree software (40).

### Antimicrobial susceptibility testing

The susceptibility of isolates to 18 antibiotics was determined using the broth microdilution method according to Clinical and Laboratory Standards Institute (CLSI) guidelines (41). The antibiotics tested included amoxicillin-clavulanic acid, cefoxitin, ceftiofur, clindamycin, doxycycline, enrofloxacin, erythromycin, florfenicol, gentamicin, linezolid, ofloxacin, oxacillin, penicillin, sulfamethoxazole, sulfisoxazole, tiamulin, tilmicosin, and vancomycin. The standard reference strain *S. aureus* ATCC 29213 was utilized as the quality control strain. The results of the quality control strains were consistent with the reference range established by CLSI standards, thereby ensuring the reliability of the antimicrobial susceptibility testing results. Strains of *S. aureus* that demonstrate resistance to at least three distinct classes of antibiotics are categorized as MDR strains.

### MRSA identification

The strains were tentatively categorized as MRSA based on their resistance to oxacillin. Furthermore, WGS was employed to detect the *mecA* gene, which is linked to methicillin resistance, thereby validating the presence of MRSA strains at the genotypic level.

### Antimicrobial resistance gene and virulence gene detection

The sequencing data were submitted individually to the Comprehensive Antibiotic Resistance Database (CARD, https://card.mcmaster.ca/analyze/rgi) and the Virulence Factor Database (VFDB, http://www.mgc.ac.cn/cgi-bin/VFs/v5/main.cgi). By analyzing and comparing the results, it can be inferred that the isolates harbor both antimicrobial resistance genes and virulence genes. To further validate the prediction of antibiotic resistance genes, all samples were reanalyzed using ResFinder (v4.6.0) to provide a more precise prediction of antibiotic resistance.

### Phylogenetic analysis of ST59 isolates

A total of 48 ST59 *S. aureus* genomes were obtained from the NCBI database for phylogenetic analysis. To delineate the genetic relationships among the ST9 isolates, a phylogenetic tree was constructed based on SNPs using Snippy (v4.4.5) (https://github.com/tseemann/snippy) with the ST59-MRSA strain SA957 (BioSample accession: SAMN00996491) as a reference strain. The phylogenetic tree was annotated and visualized by the iTOL software.

### Data analysis and visualization

The Pearson algorithm of IBM SPSS Statistics version 26 (IBM SPSS Inc., Chicago, USA) was used to calculate the correlations among the virulence genes carried by the isolates. GraphPad Prism software (v5.01; GraphPad, San Diego, California, USA) was employed to create the graphs.

## Data Availability

The supplementary data contains information regarding the antimicrobial susceptibility profiles and whole genome sequences of the isolated strains. All isolates' genomes sequenced in this study are available under the NCBI BioProject: PRJNA1101705.

## SUPPLEMENTAL MATERIAL

Tables S1 to S7 spectrum.02800-24-s0001.doc Whole genome information and sample source information of 77 *Staphylococcus aureus* isolates.

ASM does not own the copyrights to Supplemental Material that may be linked to, or accessed through, an article. The authors have granted ASM a non-exclusive, world-wide license to publish the Supplemental Material files. Please contact the corresponding author directly for reuse.
