# Supplementary material for: Antimicrobial resistance and molecular characteristics of bovine mastitis-associated methicillin-resistant Staphylococcus aureus: potential for cross-species transmission of ST59-MRSA
Source: Microbiol Spectr. 2025 May 21;13(7):e02800-24. doi: 10.1128/spectrum.02800-24 (PMC12210863; doi:10.1128/spectrum.02800-24)
Supplement: Tables S1 to S7 — Whole genome information and sample source information of 77 Staphylococcus aureus isolates. [file spectrum.02800-24-s0001.doc]

**Supplementary Data**

Antimicrobial resistance and molecular characteristics of bovine mastitis-associated methicillin-resistant *Staphylococcus aureus*: potential for cross-species transmission of ST59-MRSA

Wen Sun^1#^, Jiayi Liu^1#^, Shuangshuang Li^1^, Xiaoman Zhu^1^, Xiangyun Wu^1^, Baojing Dou^1^, Xiaomin Pang^1^, Keke Tian^1^, Peipei Wang^3^, Haihong Hao^1,2^, Yulian Wang^1,2*^

1 College of Animal Science and Veterinary Medicine, Huazhong Agricultural University, Wuhan 430070, P. R. China

2 National Reference Laboratory of Veterinary Drug Residues, Huazhong Agricultural University, Wuhan 430070, P. R. China

3 Shenzhen Kingsino Technology Co., LTD, Shenzhen 518107, P. R. China

* Corresponding author:

Yulian Wang (Y. Wang), Mail address: National Reference Laboratory of Veterinary Drug Residues, Huazhong Agricultural University, Wuhan 430070, China; Tel: +86-27-87287140; Fax: +86-27-87672232; E-mail: wangyulian@mail.hzau.edu.cn

**Tables**

**Table S1.** The prevalence of *S. aureus* in milk from eight farms in three provinces.

| **Region** | **Farm** | **No. samples** | **No. isolates** | **Proportion (%)** |
| --- | --- | --- | --- | --- |
| Hubei | A | 41 | 14 | 34.1 |
|  | B | 93 | 14 | 15.1 |
| Hunan | C | 39 | 10 | 25.6 |
|  | D | 40 | 10 | 25.0 |
|  | E | 52 | 9 | 17.3 |
| Jiangxi | F | 56 | 10 | 17.9 |
|  | G | 19 | 5 | 26.3 |
|  | H | 24 | 5 | 20.8 |
| Total | | 364 | 72 | 21.2 |

**Table S2**. Antimicrobial resistance phenotypes of 77 *S. aureus* strains.

| **Antimicrobial category** | **Number of isolates (%)** | | |
| --- | --- | --- | --- |
|  | **Overall (n = 77)** | **MSSA (n = 63)** | **MRSA (n = 14)** |
| Penicillin (PEN) | 75 (97.4) | 61 (96.8) | 14 (100.0) |
| Amoxicillin-clavulanic acid (AMC) | 46 (59.7) | 32 (50.8) | 14 (100.0) |
| Ceftiofur (TIO) | 14 (18.2) | 7 (11.1) | 7 (50.0) |
| Cefoxitin (FOX) | 14 (18.2) | 0 (0.0) | 14 (100) |
| Oxacillin (OXA) | 10 (13.0) | 2 (3.2) | 8 (57.1) |
| Erythromycin (ERY) | 25 (32.5) | 12 (19.0) | 13 (92.9) |
| Timicosin (TIL) | 13 (16.9) | 9 (14.3) | 4 (28.6) |
| Clindamycin (CLI) | 23 (29.9) | 12 (19.0) | 11 (78.6) |
| Enrofloxacin (ENR) | 29 (37.7) | 23 (36.5) | 6 (42.9) |
| Ofloxacin (OFX) | 11 (14.3) | 7 (11.1) | 4 (28.6) |
| Sulfisoxazole (SOX) | 5 (6.5) | 0 (0.0) | 5 (35.7) |
| Sulfamethoxazole (SXT) | 9 (11.7) | 5 (7.9) | 4 (42.9) |
| Vancomycin (VAN) | 0 (0.0) | 0 (0.0) | 0 (0.0) |
| Doxycycline (DOX) | 0 (0.0) | 0 (0.0) | 0 (0.0) |
| Florfenicol (FFC) | 8 (10.4) | 3 (4.8) | 5 (35.7) |
| Tiamulin (TIA) | 7 (9.1) | 3 (4.8) | 4 (28.6) |
| Gentamicin (GEN) | 15 (19.5) | 8 (12.7) | 7 (50.0) |
| Linezolid (LZ) | 1 (1.3) | 0 (0.0) | 1 (7.1) |

**Table S3.** Prevalence of resistance genes among 77 *S. aureus* strains.

| **Antimicrobial category** | **Antimicrobial resistance genes** | **Number of isolates (%)** | | |
| --- | --- | --- | --- | --- |
|  |  | **Overall**  **(n = 77)** | **MSSA**  **(n = 63)** | **MRSA**  **(n = 14)** |
| Aminoglycosides | *aac(6')-aph(2'')* | 11 (14.3) | 4 (6.3) | 7 (50.0) |
|  | *ant(4')-Ia* | 10 (13.0) | 5 (7.9) | 5 (35.7) |
|  | *ant(6)-Ia* | 13 (16.9) | 2 (3.2) | 11 (78.6) |
|  | *aph(2'')-Ia* | 5 (6.5) | 5 (100.0) | 0 (0.0) |
|  | *aph(3')-III* | 5 (6.5) | 0 (0.0) | 5 (100.0) |
| β-lactams | *blaZ* | 66 (85.7) | 53 (84.1) | 13 (92.9) |
|  | *mecA* | 15 (19.5) | 1 (1.6) | 14 (100.0) |
| lincosamides | *erm*(B) | 10 (13.0) | 4 (6.3) | 6 (42.9) |
|  | *erm*(C) | 13 (16.9) | 10 (15.9) | 3 (21.4) |
|  | *lsa*(E) | 8 (10.4) | 3 (4.8) | 5 (35.7) |
| Amphenicols | *fexA* | 8 (10.4) | 3 (4.8) | 5 (35.7) |
|  | *cat* (pC233) | 2 (2.6) | 0 (0.0) | 2 (14.3) |
| Tetracyclines | *tet*(K) | 16 (20.8) | 13 (20.6) | 3 (21.4) |
|  | *tet*(L) | 7 (9.1) | 2 (3.2) | 5 (35.7) |
|  | *tet*(M) | 1 (1.3) | 1 (1.6) | 0 (0.0) |
| Folate pathway antagonist | *dfrE* | 9 (11.7) | 9 (14.3) | 0 (35.7) |
|  | *dfrG* | 8 (10.4) | 3 (4.8) | 5 (35.7) |
| Macrolides | *erm*(B) | 10 (13.0) | 4 (6.3) | 6 (42.9) |
|  | *erm*(C) | 13 (16.9) | 10 (15.9) | 3 (21.4) |
|  | *erm*(T) | 1 (1.3) | 1 (1.6) | 0 (0.0) |

**Table S4.** Prevalence of virulence genes among 77 *S. aureus* strains.

| **Virulence factors** | **Virulence genes** | **Number of isolates (%)** | | |
| --- | --- | --- | --- | --- |
|  |  | **Overall (n = 77)** | **MSSA (n = 63)** | **MRSA (n = 14)** |
| Adherence | *atl* | 76 (98.7) | 62 (98.4) | 14 (100.0) |
|  | *ebh* | 48 (62.3) | 37 (58.7) | 11 (78.6) |
|  | *clfA* | 21 (27.3) | 20 (31.7) | 1 (7.1) |
|  | *clfB* | 7 (9.1) | 7 (11.1) | 0 (0.0) |
|  | *cna* | 17 (22.1) | 17 (27.0) | 0 (0.0) |
|  | *ebp* | 77 (100.0) | 63 (100.0) | 14 (100.0) |
|  | *eap/map* | 9 (11.7) | 7 (10.4) | 2 (14.3) |
|  | *efb* | 77 (100.0) | 63 (100.0) | 14 (100.0) |
|  | *fnbA* | 73 (94.8) | 59 (93.7) | 14 (100.0) |
|  | *fnbB* | 70 (90.9) | 57 (90.5) | 13 (92.9) |
|  | *icaA* | 77 (100.0) | 63 (100.0) | 14 (100.0) |
|  | *icaB* | 75 (97.4) | 61 (96.8) | 14 (100.0) |
|  | *icaC* | 77 (100.0) | 63 (100.0) | 14 (100.0) |
|  | *icaD* | 26 (33.8) | 23 (36.5) | 3 (21.4) |
|  | *icaR* | 77 (100.0) | 63 (100.0) | 14 (100.0) |
|  | *sdrC* | 75 (97.4) | 61 (96.8) | 14 (100.0) |
|  | *sdrD* | 61 (79.2) | 48 (76.2) | 13 (92.9) |
|  | *sdrE* | 70 (90.9) | 57 (90.5) | 13 (92.9) |
|  | *spa* | 72 (93.5) | 58 (92.1) | 14 (100.0) |
|  | *EfaA* | 1 (1.3) | 1 (1.6) | 0 (0.0) |
|  | *groEL* | 1 (1.3) | 1 (1.6) | 0 (0.0) |
|  | *lap* | 1 (1.3) | 1 (1.6) | 0 (0.0) |
|  | *flmH* | 1 (1.3) | 1 (1.6) | 0 (0.0) |
|  | *slrA* | 1 (1.3) | 1 (1.6) | 0 (0.0) |
|  | *plr/gapA* | 1 (1.3) | 1 (1.6) | 0 (0.0) |
| Enzyme | *sspB* | 77 (100.0) | 63 (100.0) | 14 (100.0) |
|  | *sspC* | 74 (96.1) | 60 (95.2) | 14 (100.0) |
|  | *hysA* | 77 (100.0) | 63 (100.0) | 14 (100.0) |
|  | *geh* | 77 (100.0) | 63 (100.0) | 14 (100.0) |
|  | *lip* | 77 (100.0) | 63 (100.0) | 14 (100.0) |
|  | *sspA* | 77 (100.0) | 63 (100.0) | 14 (100.0) |
|  | *splA* | 51 (66.2) | 49 (77.8) | 2 (14.3) |
|  | *splB* | 51 (66.2) | 49 (77.8) | 2 (14.3) |
|  | *splC* | 51 (66.2) | 49 (77.8) | 2 (14.3) |
|  | *splD* | 48 (62.3) | 46 (73.0) | 2 (14.3) |
|  | *splE* | 35 (45.5) | 33 (52.4) | 2 (14.3) |
|  | *splF* | 40 (51.9) | 38 (60.3) | 2 (14.3) |
|  | *coa* | 69 (89.6) | 55 (87.3) | 14 (100.0) |
|  | *sak* | 41 (53.2) | 33 (52.3) | 8 (57.1) |
|  | *nuc* | 77 (100.0) | 63 (100.0) | 14 (100.0) |
|  | *eno* | 1 (1.3) | 1 (1.6) | 0 (0.0) |
| Immune evasion | *adsA* | 74 (96.1) | 61 (96.8) | 13 (92.9) |
|  | *chp* | 29 (37.7) | 21 (33.3) | 8 (57.1) |
|  | *scn* | 51 (66.2) | 39 (61.9) | 12 (85.7) |
|  | *sbi* | 77 (100.0) | 63 (100.0) | 14 (100.0) |
|  | *acpXL* | 1 (1.3) | 1 (1.6) | 0 (0.0) |
|  | *gtaB* | 1 (1.3) | 1 (1.6) | 0 (0.0) |
| Secretion system | *esaA* | 77 (100.0) | 63 (100.0) | 14 (100.0) |
|  | *esaB* | 61 (79.2) | 48 (76.2) | 13 (92.9) |
|  | *esaD* | 57 (74.0) | 49 (77.8) | 8 (57.1) |
|  | *esaE* | 57 (74.0) | 49 (77.8) | 8 (57.1) |
|  | *esaG* | 77 (100.0) | 63 (100.0) | 14 (100.0) |
|  | *essA* | 73 (94.8) | 59 (93.6) | 14 (100.0) |
|  | *essB* | 77 (100.0) | 63 (100.0) | 14 (100.0) |
|  | *essC* | 66 (85.7) | 52 (82.5) | 14 (100.0) |
|  | *esxA* | 77 (100.0) | 63 (100.0) | 14 (100.0) |
|  | *esxB* | 58 (75.3) | 50 (79.4) | 8 (57.1) |
|  | *esxC* | 57 (74.0) | 49 (77.8) | 8 (57.1) |
|  | *esxD* | 46 (59.7) | 38 (60.3) | 8 (57.1) |
|  | *clpB* | 1 (1.3) | 1 (1.6) | 0 (0.0) |
| Toxin | *hly/hla* | 77 (100.0) | 63 (100.0) | 14 (100.0) |
|  | *hlb* | 1 (1.3) | 1 (1.6) | 0 (0.0) |
|  | *hld* | 66 (85.7) | 52 (82.5) | 14 (100.0) |
|  | *sea* | 12 (15.6) | 7 (11.1) | 5 (35.7) |
|  | *seb* | 19 (20.9) | 13 (19.4) | 6 (35.7) |
|  | *sec* | 6 (7.8) | 5 (7.9) | 1 (7.1) |
|  | *seg* | 33 (42.9) | 30 (47.6) | 3 (21.4) |
|  | *seh* | 3 (3.9) | 3 (4.8) | 0 (0.0) |
|  | *sei* | 11 (14.3) | 11 (17.5) | 0 (0.0) |
|  | *yent1* | 5 (6.5) | 5 (7.9) | 0 (0.0) |
|  | *yent2* | 23 (29.9) | 20 (31.7) | 3 (21.4) |
|  | *selk* | 34 (44.2) | 25 (39.7) | 9 (64.3) |
|  | *sell* | 4 (5.2) | 4 (6.3) | 0 (0.0) |
|  | *selm* | 25 (32.5) | 22 (34.9) | 3 (21.4) |
|  | *seln* | 34 (44.2) | 31 (49.2) | 3 (21.4) |
|  | *selo* | 37 (48.1) | 32 (50.8) | 5 (35.7) |
|  | *selp* | 2 (2.6) | 2 (3.2) | 0 (0.0) |
|  | *selq* | 20 (26.0) | 9 (14.3) | 11 (78.6) |
|  | *selu* | 11 (14.3) | 11 (17.5) | 0 (0.0) |
|  | *set11* | 16 (20.8) | 161 (25.4) | 0 (0.0) |
|  | *set12* | 3 (3.9) | 2 (3.2) | 1 (7.1) |
|  | *set13* | 26 (33.8) | 21 (33.3) | 5 (35.7) |
|  | *set15* | 18 (23.4) | 18 (28.6) | 0 (0.0) |
|  | *set16* | 43 (55.8) | 41 (65.1) | 2 (14.3) |
|  | *set17* | 17 (22.1) | 12 (19.0) | 5 (35.7) |
|  | *set18* | 52 (67.5) | 38 (60.3) | 14 (100.0) |
|  | *set19* | 42 (54.5) | 30 (47.6) | 12 (85.7) |
|  | *set1* | 6 (7.8) | 6 (9.5) | 0 (0.0) |
|  | *set20* | 2 (2.6) | 0 (0.0) | 2 (14.3) |
|  | *set21* | 23 (29.9) | 15 (23.8) | 8 (57.1) |
|  | *set22* | 54 (70.1) | 41 (62.1) | 13 (92.9) |
|  | *set23* | 6 (7.8) | 6 (9.5) | 0 (0.0) |
|  | *set24* | 30 (39.0) | 24 (38.1) | 6 (42.9) |
|  | *set25* | 50 (64.9) | 44 (69.8) | 6 (42.9) |
|  | *set26* | 58 (75.3) | 45 (71.4) | 13 (92.9) |
|  | *set2* | 6 (7.8) | 6 (9.5) | 0 (0.0) |
|  | *set30* | 21 (27.3) | 9 (14.3) | 12 (85.7) |
|  | *set31* | 25 (32.5) | 16 (25.4) | 9 (64.3) |
|  | *set33* | 10 (13.0) | 10 (15.9) | 0 (0.0) |
|  | *set34* | 69 (89.6) | 57 (90.5) | 12 (85.7) |
|  | *set36* | 1 (1.3) | 0 (0.0) | 1 (7.1) |
|  | *set37* | 50 (64.9) | 37 (58.7) | 13 (92.9) |
|  | *set38* | 13 (16.9) | 10 (15.9) | 3 (21.4) |
|  | *set39* | 21 (27.3) | 13 (20.6) | 8 (57.1) |
|  | *set3* | 6 (7.8) | 6 (9.5) | 0 (0.0) |
|  | *set40* | 1 (1.3) | 0 (0.0) | 1 (7.1) |
|  | *set4* | 6 (7.8) | 6 (9.5) | 0 (0.0) |
|  | *set5* | 6 (7.8) | 6 (9.5) | 0 (0.0) |
|  | *set6* | 13 (16.9) | 13 (20.6) | 0 (0.0) |
|  | *set7* | 35 (45.5) | 35 (55.6) | 0 (0.0) |
|  | *set8* | 8 (10.4) | 8 (12.7) | 0 (0.0) |
|  | *set9* | 5 (6.5) | 5 (7.9) | 0 (0.0) |
|  | *hlgA* | 77 (100.0) | 63 (100.0) | 14 (100.0) |
|  | *hlgB* | 77 (100.0) | 63 (100.0) | 14 (100.0) |
|  | *hlgC* | 77 (100.0) | 63 (100.0) | 14 (100.0) |
|  | *lukM* | 11 (14.3) | 11 (17.5) | 0 (0.0) |
|  | *lukD* | 50 (64.9) | 48 (76.2) | 2 (14.3) |
|  | *lukF-PV* | 3 (3.9) | 2 (3.2) | 1 (7.1) |
|  | *lukS-PV* | 3 (3.9) | 2 (3.2) | 1 (7.1) |
|  | *tsst* | 1 (1.3) | 1 (1.6) | 0 (0.0) |
|  | *cylR2* | 2 (2.6) | 2 (3.2) | 0 (0.0) |

**Table S5.** Characteristics of the genomes of MRSA isolates.

| **Isolates** | **MLST** | ***spa* type** | **SCC*mec* type** | **Resistance gene profiles** | **Virulence gene profiles** |
| --- | --- | --- | --- | --- | --- |
| SA-HN20e106 | 9 | t899 | XII | *aac(6′)-aph(2′′)*; *ant(4′)-Ia*; *ant(6)-Ia*; *blaZ*; *dfrG*; *erm*(C); *fexA*; *lsaE*; *mecA*; *tet*(L) | *atl*; *ebh*; *ebp*; *efb*; *fnbA*; *fnbB*; *icaA*; *icaB*; *icaC*; *icaD*; *icaR*; *sdrC*; *sdrD*; *sdrE*; *spa*; *sspB*; *sspC*; *hysA*; *geh*; *lip*; *sspA*; *coa*; *nuc*; *adsA*; *scn*; *sbi*; *esaA*; *esaB*; *esaD*; *esaE*; *esaG*; *essA*; *essB*; *essC*; *esxA*; *esxB*; *esxC*; *esxD*; *hly/hla*; *hld*; *seg*; *yent2*; *selk*; *selm*; *seln*; *selo*; *selq*; *set13*; *set17*; *set18*; *set19*; *set21*; *set22*; *set26*; *set30*; *set34*; *set37*; *set39*; *hlgA*; *hlgB*; *hlgC* |
| SA-HN20e112 | 9 | t899 | XII | *aac(6′)-aph(2′′)*; *ant(4′)-Ia*; *ant(6)-Ia*; *blaZ*; *dfrG*; *erm*(C); *fexA*; *lsaE*; *mecA*; *tet*(L) | *atl*; *ebh*; *ebp*; *efb*; *fnbA*; *icaA*; *icaB*; *icaC*; *icaR*; *sdrC*; *sdrD*; *sdrE*; *spa*; *sspB*; *sspC*; *hysA*; *geh*; *lip*; *sspA*; *coa*; *sak*; *nuc*; *adsA*; *chp*; *sbi*; *esaA*; *esaB*; *esaG*; *essA*; *essB*; *essC*; *esxA*; *hly/hla*; *hld*; *sea*; *seb*; *selk*; *selq*; *set18*; *set19*; *set22*; *set24*; *set25*; *set26*; *set30*; *set31*; *set34*; *set37*; *hlgA*; *hlgB*; *hlgC*; *lukF-PV*; *lukS-PV* |
| SA-HN20e113 | 9 | t899 | XII | *aac(6′)-aph(2′′)*; *ant(4′)-Ia*; *ant(6)-Ia*; *blaZ*; *dfrG*; *erm*(C); *fexA*; *lsaE*; *mecA*; *tet*(L) | *atl; ebh; ebp; efb; fnbA; fnbB; icaA; icaB; icaC; icaR; sdrC; sdrD; sdrE; spa; sspB; sspC; hysA; geh; lip; sspA; coa; nuc; adsA; scn; sbi; esaA; esaB; esaD; esaE; esaG; essA; essB; essC; esxA; esxB; esxC; esxD; hly/hla; hld; sec; seg; vent2; selk; selm; seln; selo; selq; set13; set17; set18; set19; set21; set22; set26; set30; set34; set37; set39; hlgA; hlgB; hlgC* |
| SA-JX20e150 | 9 | t899 | XII | *aac(6′)-aph(2′′)*; *ant(4′)-Ia*; *ant(6)-Ia*; *blaZ*; *dfrG*; *erm*(C); *fexA*; *lsaE*; *mecA*; *tet*(L) | *atl*; *ebh*; *ebp*; *efb*; *fnbA*; *fnbB*; *icaA*; *icaB*; *icaC*; *icaR*; *sdrC*; *sdrD*; *sdrE*; *spa*; *sspB*; *sspC*; *hysA*; *geh*; *lip*; *sspA*; *coa*; *nuc*; *adsA*; *scn*; *sbi*; *esaA*; *esaB*; *esaD*; *esaE*; *esaG*; *essA*; *essB*; *essC*; *esxA*; *esxB*; *esxC*; *esxD*; *hly/hla*; *hld*; *selo*; *selq*; *set13*; *set17*; *set18*; *set19*; *set20*; *set21*; *set22*; *set26*; *set30*; *set37*; *set39*; *hlgA*; *hlgB*; *hlgC* |
| SA-HN20e107 | 59 | t437 | IV.a | *ant(6)-Ia*; *aph(3')-III*; *blaZ*; *erm*(B); *mecA* | *atl*; *ebh*; *ebp*; *efb*; *fnbA*; *fnbB*; *icaA*; *icaB*; *icaC*; *icaR*; *sdrC*; *sdrD*; *sdrE*; *spa*; *sspB*; *sspC*; *hysA*; *geh*; *lip*; *sspA*; *coa*; *nuc*; *adsA*; *scn*; *sbi*; *esaA*; *esaB*; *esaD*; *esaE*; *esaG*; *essA*; *essB*; *essC*; *esxA*; *esxB*; *esxC*; *esxD*; *hly*/*hla*; *hld*; *selo*; *selq*; *set12*; *set13*; *set17*; *set18*; *set19*; *set20*; *set21*; *set22*; *set26*; *set30*; *set39*; *hlgA*; *hlgB*; *hlgC* |
| SA-JX20e146 | 59 | t437 | IV.a | *ant(6)-Ia*; *aph(3')-III*; *blaZ*; *erm*(B); *mecA*; *tet*(K) | *atl*; *ebh*; *ebp*; *efb*; *fnbA*; *fnbB*; *icaA*; *icaB*; *icaC*; *icaD*; *icaR*; *sdrC*; *sdrD*; *sdrE*; *spa*; *sspB*; *sspC*; *hysA*; *geh*; *lip*; *sspA*; *splA*; *splB*; *splC*; *splD*; *splE*; *splF*; *coa*; *sak*; *nuc*; *adsA*; *chp*; *scn*; *sbi*; *esaA*; *esaB*; *seaD*; *esaE*; *esaG*; *essA*; *essB*; *essC*; *esxA*; *esxB*; *esxC*; *esxD*; *hly*/*hla*; *hld*; *sea*; *set16*; *set18*; *set19*; *set21*; *set22*; *set26*; *set31*; *set34*; *set37*; *set38*; *set39*; *hlgA*; *hlgB*; *hlgC*; *lukD* |
| SA-JX20e147 | 59 | t437 | IV.g | *aac(6′)-aph(2′′)*; *ant(6)-Ia*; *aph(3')-III*; *blaZ*; *cat(pC233)*; *erm*(B); *mecA*; *tet*(K) | *atl*; *ebh*; *clfA*; *ebp*; *efb*; *fnbA*; *fnbB*; *icaA*; *icaB*; *icaC*; *icaR*; *sdrC*; *spa*; *sspB*; *sspC*; *hysA*; *geh*; *lip*; *sspA*; *coa*; *nuc*; *sbi*; *esaA*; *esaD*; *esaE*; *esaG*; *essA*; *essB*; *essC*; *esxA*; *esxB*; *esxC*; *esxD*; *hly*/*hla*; *hld*; *set18*; *set21*; *set30*; *set31*; *set34*; *set36*; *set37*; *set38*; *set39*; *set40*; *hlgA*; *hlgB*; *hlgC* |
| SA-JX20e148 | 59 | t437 | IV.g | *aac(6′)-aph(2′′)*; *ant(6)-Ia*; *aph(3')-III*; *cat(pC233)*; *erm*(B); *mecA* | *atl*; *ebp*; *efb*; *fnbA*; *fnbB*; *icaA*; *icaB*; *icaC*; *icaR*; *sdrC*; *sdrD*; *sdrE*; *spa*; *sspB*; *sspC*; *hysA*; *geh*; *lip*; *sspA*; *coa*; *sak*; *nuc*; *adsA*; *chp*; *scn*; *sbi*; *esaA*; *esaB*; *esaG*; *essA*; *essB*; *essC*; *esxA*; *hly*/*hla*; *hld*; *seb*; *selk*; *selq*; *set18*; *set19*; *set22*; *set24*; *set25*; *set26*; *set30*; *set31*; *set34*; *set37*; *hlgA*; *hlgB*; *hlgC* |
| SA-JX20e149 | 59 | t437 | IV.a | *ant(6)-Ia*; *aph(3')-III*; *blaZ*; *erm*(B); *mecA* | *atl*; *ebh*; *ebp*; *efb*; *fnbA*; *fnbB*; *icaA*; *icaB*; *icaC*; *icaD*; *icaR*; *sdrC*; *sdrD*; *sdrE*; *spa*; *sspB*; *sspC*; *hysA*; *geh*; *lip*; *sspA*; *splA*; *splB*; *splC*; *splD*; *splE*; *splF*; *coa*; *sak*; *nuc*; *adsA*; *chp*; *scn*; *sbi*; *esaA*; *esaB*; *seaD*; *esaE*; *esaG*; *essA*; *essB*; *essC*; *esxA*; *esxB*; *esxC*; *esxD*; *hly*/*hla*; *hld*; *sea*; *set16*; *set18*; *set19*; *set21*; *set22*; *set26*; *set31*; *set34*; *set37*; *set38*; *set39*; *hlgA*; *hlgB*; *hlgC*; *lukD* |
| SA-HN20e122 | 88 | t3622 | IV.c | *aph(3')-III*; *blaZ*; *erm*(C); *mecA* | *atl*; *ebp*; *efb*; *fnbA*; *fnbB*; *icaA*; *icaB*; *icaC*; *icaR*; *sdrC*; *sdrD*; *sdrE*; *spa*; *sspB*; *sspC*; *hysA*; *geh*; *lip*; *sspA*; *coa*; *sak*; *nuc*; *adsA*; *chp*; *scn*; *sbi*; *esaA*; *esaB*; *esaG*; *essA*; *essB*; *essC*; *esxA*; *hly*/*hla*; *hld*; *seb*; *selk*; *selq*; *set18*; *set19*; *set22*; *set24*; *set25*; *set26*; *set30*; *set31*; *set34*; *set37*; *hlgA*; *hlgB*; *hlgC* |
| SA-HN20e127 | 88 | t3622 | IV.c | *blaZ*; *erm*(C); *mecA* | *atl*; *ebh*; *ebp*; *eap*/*map*; *efb*; *fnbA*; *fnbB*; *icaA*; *icaB*; *icaC*; *icaR*; *sdrC*; *sdrD*; *sdrE*; *spa*; *sspB*; *sspC*; *hysA*; *geh*; *lip*; *sspA*; *coa*; *sak*; *nuc*; *adsA*; *chp*; *scn*; *sbi*; *esaA*; *esaB*; *esaG*; *essA*; *essB*; *essC*; *esxA*; *hly*/*hla*; *hld*; *sea*; *seb*; *selk*; *selq*; *set18*; *set19*; *set22*; *set24*; *set25*; *set26*; *set30*; *set31*; *set34*; *set37*; *hlgA*; *hlgB*; *hlgC* |
| SA-HN20e123 | 630 | t14066 | V | *blaZ*; *mecA* | *atl*; *ebh*; *ebp*; *eap*/*map*; *efb*; *fnbA*; *fnbB*; *icaA*; *icaB*; *icaC*; *icaR*; *sdrC*; *sdrD*; *sdrE*; *spa*; *sspB*; *sspC*; *hysA*; *geh*; *lip*; *sspA*; *coa*; *sak*; *nuc*; *adsA*; *chp*; *scn*; *sbi*; *esaA*; *esaB*; *esaG*; *essA*; *essB*; *essC*; *esxA*; *hly*/*hla*; *hld*; *sea*; *seb*; *selk*; *selq*; *set18*; *set19*; *set22*; *set24*; *set25*; *set26*; *set30*; *set31*; *set34*; *set37*; *hlgA*; *hlgB*; *hlgC* |
| SA-HN20e124 | 4513 | t437 | IV.a | *ant(6)-Ia*; *aph(3')-III*; *blaZ*; *erm*(B); *mecA*; *tet*(K) | *atl*; *ebp*; *efb*; *fnbA*; *fnbB*; *icaA*; *icaB*; *icaC*; *icaR*; *sdrC*; *sdrD*; *sdrE*; *spa*; *sspB*; *sspC*; *hysA*; *geh*; *lip*; *sspA*; *coa*; *sak*; *nuc*; *adsA*; *chp*; *scn*; *sbi*; *esaA*; *esaB*; *esaG*; *essA*; *essB*; *essC*; *esxA*; *hly*/hla; *hld*; *seb*; *selk*; *selq*; *set18*; *set19*; *set22*; *set24*; *set25*; *set26*; *set30*; *set31*; *set34*; *set37*; *hlgA*; *hlgB*; *hlgC* |
| SA-HN20e111 | 7181 | t899 | XII | *aac(6′)-aph(2′′)*; *ant(4′)-Ia*; *ant(6)-Ia*; *blaZ*; *dfrG*; *erm*(C); *fexA*; *lsaE*; *mecA*; *tet*(L) | *atl*; *ebh*; *ebp*; *efb*; *fnbA*; *fnbB*; *icaA*; *icaB*; *icaC*; *icaR*; *sdrC*; *sdrD*; *sdrE*; *spa*; *sspB*; *sspC*; *hysA*; *geh*; *lip*; *sspA*; *coa*; *nuc*; *adsA*; *scn*; *sbi*; *esaA*; *esaB*; *esaD*; *esaE*; *esaG*; *essA*; *essB*; *essC*; *esxA*; *esxB*; *esxC*; *esxD*; *hly*/*hla*; *hld*; *seg*; *yent2*; *selk*; *selm*; *seln*; *selo*; *selq*; *set13*; *set17*; *set18*; *set21*; *set22*; *set26*; *set30*; *set34*; *set37*; *set39*; *hlgA*; *hlgB*; *hlgC* |

**Table S6.** Metadata of the international ST59 isolates

| **Isolate** | **Location** | **Host** | **MRSA/MSSA** | **SCC*mec*** | **NCBI assembly** |
| --- | --- | --- | --- | --- | --- |
| M013 | China | Homo sapiens | MRSA | V.b | GCF_000237125.2 |
| SA957 | China | Homo sapiens | MRSA | V.b | GCF_000470845.1 |
| SA268 | China | Homo sapiens | MRSA | IV | GCF_000737615.1 |
| HZW450 | China | Homo sapiens | MRSA | V.b | GCF_002442975.1 |
| VGC1 | China | Homo sapiens | MRSA | V.b | GCF_008330045.1 |
| SAW1 | China | Homo sapiens | MRSA | IV.a | GCF_009914455.1 |
| Guangzhou-SAU071 | China | Homo sapiens | MRSA | IV.a | GCF_013046885.1 |
| ST59 | China | Homo sapiens | MRSA | IV.a | GCF_018987365.1 |
| DC.RB_015 | China | Food（Rubing Cheese） | MRSA | IV.a | GCF_020042725.1 |
| S36 | China | Homo sapiens | MRSA | V.b | GCF_020702515.2 |
| SYN | China | Homo sapiens | MSSA | / | GCF_022385275.1 |
| 697 | China | Homo sapiens | MRSA | IV.a | GCF_022494565.1 |
| GY8 | China | Homo sapiens | MRSA | IV.a | GCF_022532145.1 |
| 697R | China | induction of antibiotic | MRSA | IV.a | GCF_022682325.1 |
| SA38-SX | China | Homo sapiens | MRSA | IV.a | GCF_030533465.1 |
| SA37-SX | China | Homo sapiens | MRSA | V.b | GCF_030533495.1 |
| SA36-SX | China | Homo sapiens | MRSA | IV.a | GCF_030533515.1 |
| SA32-SX | China | Homo sapiens | MRSA | IV.a | GCF_030533605.1 |
| SA23-SX | China | Homo sapiens | MRSA | IV.a | GCF_030533805.1 |
| SA21-SX | China | Homo sapiens | MRSA | IV.a | GCF_030533855.1 |
| SA19-SX | China | Homo sapiens | MRSA | IV.a | GCF_030533895.1 |
| SA18-SX | China | Homo sapiens | MRSA | V.b | GCF_030533915.1 |
| SA16-SX | China | Homo sapiens | MSSA | / | GCF_030533955.1 |
| SA14-SX | China | Homo sapiens | MRSA | IV.a | GCF_030533995.1 |
| SA13-SX | China | Homo sapiens | MRSA | IV.a | GCF_030534015.1 |
| SA10-SX | China | Homo sapiens | MRSA | V.b | GCF_030534075.1 |
| SA09-SX | China | Homo sapiens | MRSA | IV.a | GCF_030534095.1 |
| SA02-SX | China | Homo sapiens | MRSA | V.b | GCF_030534235.1 |
| GDB8P68A | China | pig | MRSA | IV.a | GCF_018682235.1 |
| CQB3M005P | China | cow | MRSA | IV.a | GCF_011007275.1 |
| CQR3P007P | China | pig | MRSA | IV.a | GCF_011007265.1 |
| CQH3M012P | China | cow | MRSA | IV.a | GCF_011007255.1 |
| CQY3C006P | China | chicken | MRSA | IV.a | GCF_011007235.1 |
| YK046 | China | pig | MRSA | IV.a | GCF_003309045.1 |
| O331 | France | sheep | MSSA | / | GCF_004772055.1 |
| UP_1313 | Germany | Homo sapiens | MSSA |  | GCF_009912335.1 |
| 372 | Germany | Homo sapiens | MSSA | / | GCF_020388235.1 |
| 371 | Germany | Homo sapiens | MSSA | / | GCF_020388255.1 |
| 370 | Germany | Homo sapiens | MSSA | / | GCF_020388275.1 |
| 367 | Germany | Homo sapiens | MSSA | / | GCF_020388295.1 |
| 366 | Germany | Homo sapiens | MSSA | / | GCF_020388315.1 |
| 365 | Germany | Homo sapiens | MSSA | / | GCF_020388335.1 |
| HL20709 | South Korea | Homo sapiens | MRSA | V.b | GCF_013003945.1 |
| HL23187 | South Korea | Homo sapiens | MRSA | V.b | GCF_019603375.1 |
| NCCP11854 | South Korea | Homo sapiens | MSSA | / | GCF_021513355.1 |
| M3386D | Switzerland | cow | MSSA | / | GCF_006511635.1 |
| NAS_AN_130 | USA | Homo sapiens | MSSA | / | GCF_022405335.1 |
| SA40 | / | / | MRSA | IV.g | GCF_000470865.1 |

**Table S7.** Metadata of 77 *S. aureus* strains.

| **Isolate** | **Biosample** | **Genome Accession** | **Sources** |
| --- | --- | --- | --- |
| SA-BYS20e03 | SAMN41001117 | JBCGHY000000000 | Nipple milk of SCM cow |
| SA-BYS20e04 | SAMN41001118 | JBCGHZ000000000 | Nipple milk of SCM cow |
| SA-BYS20e05 | SAMN41001119 | JBCGIA000000000 | Nipple milk of SCM cow |
| SA-BYS20e06 | SAMN41001120 | JBCGIB000000000 | Nipple milk of healthy cow |
| SA-BYS20e07 | SAMN41001121 | JBCGIC000000000 | Nipple milk of SCM cow |
| SA-BYS20e08 | SAMN41001122 | JBCGID000000000 | Nipple milk of SCM cow |
| SA-BYS20e09 | SAMN41001123 | JBCGIE000000000 | Nipple milk of CM cow |
| SA-BYS20e10 | SAMN41001124 | JBCGIF000000000 | Nipple milk of CM cow |
| SA-BYS20e11 | SAMN41001125 | JBCGIG000000000 | Nipple milk of CM cow |
| SA-BYS20e12 | SAMN41001126 | JBCGIH000000000 | Nipple milk of CM cow |
| SA-BYS20e13 | SAMN41001127 | JBCGII000000000 | Nipple milk of CM cow |
| SA-BYS20e14 | SAMN41001128 | JBCGIJ000000000 | Nipple milk of CM cow |
| SA-BYS20e15 | SAMN41001129 | JBCGIK000000000 | Nipple milk of CM cow |
| SA-MJD20e54 | SAMN41001168 | JBCGJX000000000 | Nipple milk of SCM cow |
| SA-MJD20e55 | SAMN41001169 | JBCGJY000000000 | Nipple milk of healthy cow |
| SA-MJD20e56 | SAMN41001170 | JBCGJZ000000000 | Nipple milk of SCM cow |
| SA-MJD20e57 | SAMN41001171 | JBCGKA000000000 | Nipple milk of healthy cow |
| SA-MJD20e58 | SAMN41001172 | JBCGKB000000000 | Nipple milk of healthy cow |
| SA-MJD20e59 | SAMN41001173 | JBCGKC000000000 | Nipple milk of healthy cow |
| SA-MJD20e60 | SAMN41001174 | JBCGKD000000000 | Nipple milk of SCM cow |
| SA-MJD20e61 | SAMN41001175 | JBCGKE000000000 | Nipple milk of healthy cow |
| SA-MJD20e62 | SAMN41001176 | JBCGKF000000000 | Nipple milk of healthy cow |
| SA-MJD20e63 | SAMN41001177 | JBCGKG000000000 | Nipple milk of SCM cow |
| SA-MJD20e64 | SAMN41001178 | JBCGKH000000000 | Nipple milk of healthy cow |
| SA-MJD20e65 | SAMN41001179 | JBCGKI000000000 | Nipple milk of healthy cow |
| SA-MJD20e66 | SAMN41001180 | JBCGKJ000000000 | Nipple milk of healthy cow |
| SA-MJD20e67 | SAMN41001181 | JBCGKK000000000 | Nipple milk of CM cow |
| SA-MJD20e68 | SAMN41001182 | JBCGKL000000000 | Nipple milk of CM cow |
| SA-HN20e101 | SAMN41001215 | JBCGVO000000000 | Nipple milk of CM cow |
| SA-HN20e102 | SAMN41001216 | JBCGVP000000000 | Nipple milk of CM cow |
| SA-HN20e103 | SAMN41001217 | JBCGLR000000000 | Nipple milk of CM cow |
| SA-HN20e104 | SAMN41001218 | JBCGVQ000000000 | Nipple milk of CM cow |
| SA-HN20e105 | SAMN41001219 | JBCGVR000000000 | Nipple milk of CM cow |
| SA-HN20e106 | SAMN41001220 | JBCGVM000000000 | Nipple milk of CM cow |
| SA-HN20e107 | SAMN41001221 | JBCGLS000000000 | Nipple milk of CM cow |
| SA-HN20e108 | SAMN41001222 | JBCGVS000000000 | Nipple milk of CM cow |
| SA-HN20e109 | SAMN41001223 | JBCGLT000000000 | Nipple milk of CM cow |
| SA-HN20e110 | SAMN41001224 | JBCGLU000000000 | Nipple milk of CM cow |
| SA-HN20e111 | SAMN41001225 | JBCGVT000000000 | Nipple milk of CM cow |
| SA-HN20e112 | SAMN41001226 | JBCGLV000000000 | Nipple milk of CM cow |
| SA-HN20e113 | SAMN41001227 | JBCGLW000000000 | Nipple milk of CM cow |
| SA-HN20e114 | SAMN41001228 | JBCGLX000000000 | Nipple milk of CM cow |
| SA-HN20e116 | SAMN41001229 | JBCGLY000000000 | Nipple milk of CM cow |
| SA-HN20e117 | SAMN41001230 | JBCGLZ000000000 | Nipple milk of CM cow |
| SA-HN20e118 | SAMN41001231 | JBCGVU000000000 | Nipple milk of CM cow |
| SA-HN20e119 | SAMN41001232 | JBCGMA000000000 | Nipple milk of CM cow |
| SA-HN20e120 | SAMN41001233 | JBCGMB000000000 | Nipple milk of CM cow |
| SA-HN20e121 | SAMN41001234 | JBCGMC000000000 | Nipple milk of CM cow |
| SA-HN20e122 | SAMN41001235 | JBCGMD000000000 | Nipple milk of CM cow |
| SA-HN20e123 | SAMN41001236 | JBCGME000000000 | Nipple milk of CM cow |
| SA-HN20e124 | SAMN41001237 | JBCGMF000000000 | Nipple milk of CM cow |
| SA-HN20e125 | SAMN41001238 | JBCGMG000000000 | Nipple milk of CM cow |
| SA-HN20e126 | SAMN41001239 | JBCGVV000000000 | Nipple milk of CM cow |
| SA-HN20e127 | SAMN41001240 | JBCGVW000000000 | Nipple milk of CM cow |
| SA-HN20e128 | SAMN41001241 | JBCGVX000000000 | Nipple milk of CM cow |
| SA-HN20e129 | SAMN41001242 | JBCGMH000000000 | Nipple milk of CM cow |
| SA-HN20e130 | SAMN41001243 | JBCGVY000000000 | Nipple milk of CM cow |
| SA-JX20e131 | SAMN41001244 | JBCGVZ000000000 | Nipple milk of CM cow |
| SA-JX20e132 | SAMN41001245 | JBCGMI000000000 | Nipple milk of CM cow |
| SA-JX20e133 | SAMN41001246 | JBCGMJ000000000 | Nipple milk of CM cow |
| SA-JX20e134 | SAMN41001247 | JBCGMK000000000 | Nipple milk of CM cow |
| SA-JX20e135 | SAMN41001248 | JBCGML000000000 | Nipple milk of CM cow |
| SA-JX20e136 | SAMN41001249 | JBCGMM000000000 | Nipple milk of CM cow |
| SA-JX20e137 | SAMN41001250 | JBCGMN000000000 | Nipple milk of CM cow |
| SA-JX20e138 | SAMN41001251 | JBCGWA000000000 | Nipple milk of CM cow |
| SA-JX20e139 | SAMN41001252 | JBCGWB000000000 | Nipple milk of CM cow |
| SA-JX20e140 | SAMN41001253 | JBCGMO000000000 | Nipple milk of CM cow |
| SA-JX20e141 | SAMN41001254 | JBCGMP000000000 | Nipple milk of CM cow |
| SA-JX20e142 | SAMN41001255 | JBCGWC000000000 | Nipple milk of CM cow |
| SA-JX20e143 | SAMN41001256 | JBCGMQ000000000 | Nipple milk of CM cow |
| SA-JX20e144 | SAMN41001257 | JBCGMR000000000 | Nipple milk of CM cow |
| SA-JX20e145 | SAMN41001258 | JBCGWD000000000 | Nipple milk of CM cow |
| SA-JX20e146 | SAMN41001259 | JBCGMS000000000 | Nipple milk of CM cow |
| SA-JX20e147 | SAMN41001260 | JBCGMT000000000 | Nipple milk of CM cow |
| SA-JX20e148 | SAMN41001261 | JBCGWE000000000 | Nipple milk of CM cow |
| SA-JX20e149 | SAMN41001262 | JBCGMU000000000 | Nipple milk of CM cow |
| SA-JX20e150 | SAMN41001263 | JBCGMV000000000 | Nipple milk of CM cow |
